# CD44 mediates stem cell mobilization to damaged lung *via* its novel transcriptional targets, Cortactin and Survivin

**DOI:** 10.7150/ijms.33125

**Published:** 2020-01-01

**Authors:** Allal Ouhtit, Rajesh Thouta, Hatem Zayed, Rajiv L. Gaur, Augusta Fernando, Mizanur Rahman, David A. Welsh

**Affiliations:** 1 Department of Biological & Environmental Sciences, College of Arts & Sciences, Qatar University, Doha, Qatar.; 2 Department of Biomedical Sciences, College of Health and Sciences, Qatar University, Doha, Qatar; 3 Section of Pulmonary/Critical Care Medicine and Allergy/Immunology, Louisiana State University Health Sciences Center, New Orleans, LA, 70112, USA.

**Keywords:** mesenchymal stem cells, cigarette smoke, CD44, Cortactin, Survivin

## Abstract

Beyond their role in bone and lung homeostasis, mesenchymal stem cells (MSCs) are becoming popular in cell therapy. Various insults may disrupt the repair mechanisms involving MSCs. One such insult is smoking, which is a major risk factor for osteoporosis and respiratory diseases. Upon cigarette smoke-induced damage, a series of reparatory mechanisms ensue; one such mechanism involves Glycosaminoglycans (GAG). One of these GAGs, namely hyaluronic acid (HA), serves as a potential therapeutic target in lung injury. However, much of its mechanisms of action through its major receptor CD44 remains unexplored. Our previous studies have identified and functionally validated that both cortactin (CTTN: marker of motility) and Survivin (BIRC5: required for cell survival) act as novel HA/CD44-downstream transcriptional targets underpinning cell motility. Here, human MSCs were treated with “*Water-pipe*” smoke to investigate the effects of cigarette smoke condensate (CSC) on these HA-CD44 novel signaling pathways. Our results show that CSC decreased the expression of both CD44 and its downstream targets CTTN and BIRC5 in MSCs, and that HA reversed these effects. Interestingly, CSC inhibited migration and invasion of MSCs upon CD44-targeted RNAi treatment. This shows the importance of CD44-HA/CTTN and CD44-HA/BIRC5 signaling pathways in MSC motility, and further suggests that these signaling pathways may provide a novel mechanism implicated in migration of MSCs during repair of lung tissue injury. These findings suggest that one should use caution before utilizing MSC from donors with history of smoking, and further pave the way towards the development of targeted therapeutic approaches against CD44-associated diseases.

## Introduction

Cigarette smoking is a common risk factor for numerous systemic conditions, including respiratory diseases and osteoporosis. Cigarette smoke can cause DNA damage, free radical production, inflammation and lung cell injury, all of which negatively affect homeostasis of the lung 1-4. Furthermore, it disrupts various cellular mechanisms implicated in the repair of the damage 1-4.

Bone marrow mesenchymal stromal cells (MSCs) participate in the maintenance and repair of bone as well as acts as a reservoir of progenitor cells for various solid organs. This “reservoir” capacity allows it to contribute to the regeneration of mesenchymal tissues in the event of injury 1-4. In the lung, MSCs play a pivotal role in the regeneration of almost all cell types, thereby boosting repair mechanisms 1-4. Beyond their role in bone homeostasis, *ex vivo* expanded MSCs have also attracted considerable attention for their potential in cell therapy of a wide variety of pathological conditions 2-7. However, this potential of MSCs is detrimentally affected by cigarette thus reducing their therapeutic potency 8,9.

Glycosaminoglycans (GAGs) can protect against cigarette smoke-induced damage by potentiating repair process of smoke-mediated DNA damage. Indeed, when normal proteoglycan synthesis is disrupted using specific proteoglycan inhibitors, emphysematous changes and parenchymal destruction were observed 1-4. Furthermore, senescence and emphysema have been associated with a decrease in the GAG species of the lung 1-4. In particular, hyaluronan or HA has been reported to play a critical role in the repair process of lung tissue in emphysema. Reduction in HA content is also correlated with increased degradation of elastin by elastases 1-4. This is likely due to the fact that HA, along with other GAGs, can form a protective barrier that prevents access of elastases to elastin 1-4. In animal studies, intratracheal or aerosolic HA supplementation protected elastin fibers from degradation, a role that was compromised by hyaluronidase and lysozyme 1-4.

Binding HA to CD44 is involved in a multitude of functions, including cell adhesion, motility, angiogenesis and inflammation 10,11. Indeed, CD44 is one of the most abundant receptors on MSC and plays a major role in cell migration in response to chemotactic stimuli. The signaling pathways involved during HA-CD44 interaction have been mostly investigated in cancer 12. Several studies have established a positive correlation between cell invasiveness and increased expression of HA, HAS2 (HA synthase 2), HYAL2 (hyaluronidase 2), and CD44 13,14. However, the molecular pathways implicated in HA-CD44-induced migration of MSCs stimulated by nicotine, a key component of cigarette-smoke, are nascent.

We have previously identified and validated Cortactin (CTTN) 15, Survivin (BIRC5) 16, and TGF-β 17 as novel downstream transcriptional target genes in the HA/CD44 signaling pathway. Nicotine decreased CD44 in rodent bone marrow stromal cells 18. Here, we examined the role of CD44-HA interaction in migration of MSCs exposed to cigarette smoke. We provide exciting evidence implicating CD44-HA/CTTN and CD44-HA/BIRC5 in MSC cell motility. Taken together, our results suggest that these signaling pathways may provide a novel target in modulating migration of MSCs during repair of lung tissue injury.

## Results

### Establishment of the optimal dose of CSC affecting CD44

**Figure 1:**
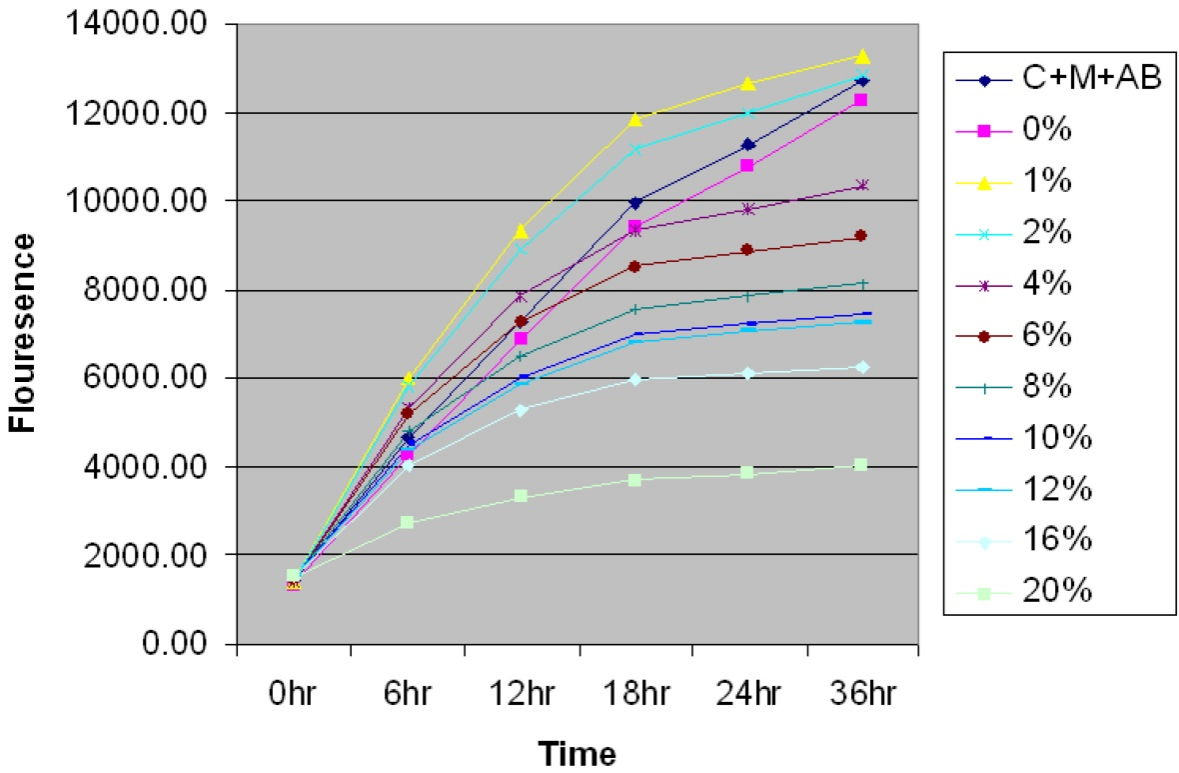
** Time- and dose-dependent effect of CSC on the proliferation of MSC.** Time and dose-dependent effect of CSC on the proliferation of MSCs. Based on the growth curves obtained, 6% CSC (~80 %) was selected as an optimal dose for further experiments.

Prior to examining the effect of CSC in MSCs, AlamarBlue® proliferation assay was utilized to determine the optimal dose of CSC required for all the experiments (Figure 1). MSC cells were seeded at 2x10^4^ cells on Costar black tissue culture plates and were allowed to attach overnight. The cells were treated with medium containing various doses of CSC. AlamarBlue® was added to all the wells. Cells were observed at appropriated time points for morphological changes and fluorometric readings were taken at excitation of 540nm and emission 610nm.

The results showed a time and dose-dependent effect of CSC on the proliferation of MSCs. Interestingly cell proliferation decreased in due course of time rather than an abrupt change indicating an induction of cell death by specific mechanism rather than a toxic effect of CSC. Based on the growth curves obtained, 6% CSC (~80 %) was selected as an optimal dose to evaluate the actions of CSC on MSCs.

### CSC decreases both CD44 RNA and protein Expression levels in MSC

**Figure 2:**
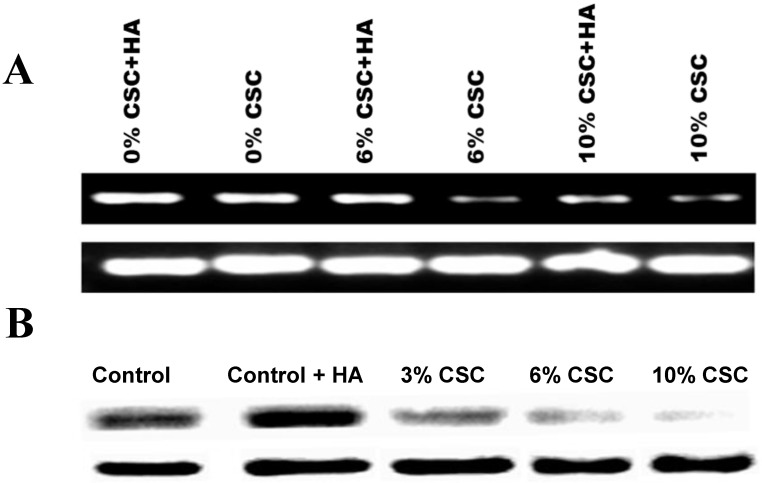
** CSC decreases both CD44 RNA and protein Expression levels in MSC. (A)** CSC decreases CD44 RNA expression levels in MSC.** (B)** CSC decreases CD44 protein expression levels in MSC. Hyaluronic Acid ameliorates CSC effect on CD44 Expression in MSC**.**

Hylorunan (100μg/ml), a known ligand of CD44 showed high expression of CD44 expression was used as a control (Figures 2A and B). The RT-PCR results showed a marked decrease in the expression of CD44 mRNA with increasing concentrations of CSC in the media (Figure 2 A). Similarly, the effect of CSC on CD44 protein expression was performed by western blot analysis showing a decrease of CD44 protein.

### HA ameliorates the CSC effect on CD44 expression in MSC

**Figure 3:**
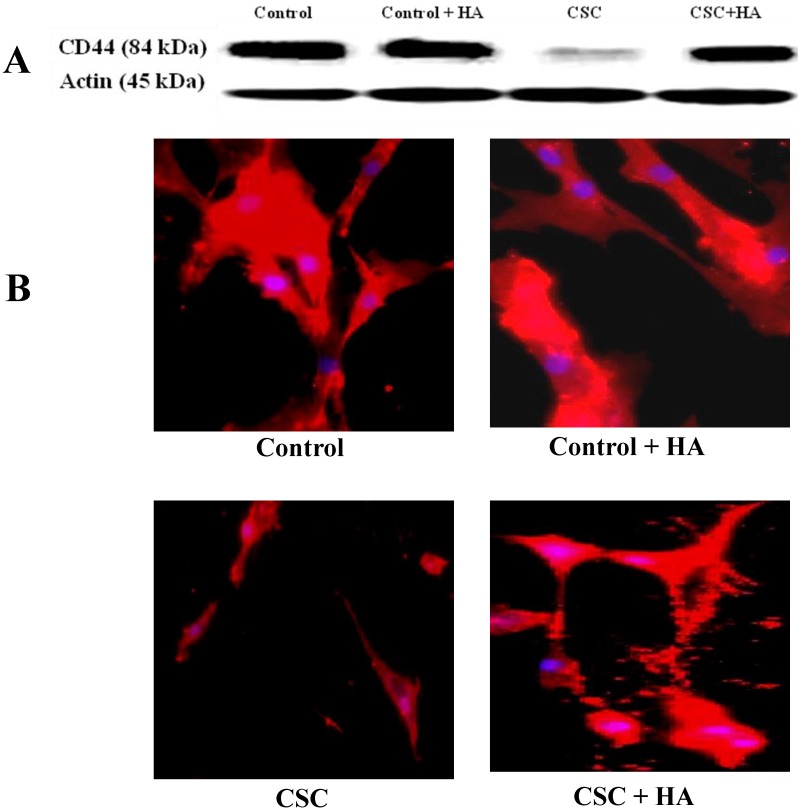
** Hyaluronan (HA) ameliorates the CSC effect on CD44 expression in MSC. (A)** While CSC decreased CD44 protein expression, treatment with HA reversed CSC's effect on CD44 protein expression. Shown are the cropped blot images representing indicated proteins, and all the gels from three separate experiments have been run under the same experimental conditions.** (C)** Immunocytochemical images showing hyaluronic acid ameliorating CSC effect on CD44 Expression in MSC.

After establishing the effect of CSC in decreasing the expression levels of CD44 both at RNA and protein levels, we tested the hypothesis whether HA could reverse this effect (Figure 3). When MSCs were treated with increasing concentrations of CSC, dose-dependent decrease in CD44 protein expression was observed (Fig. 3A). Interestingly however, treatment with HA completely abolished CSC's effect on CD44 protein expression (Fig. 3A). These results were further confirmed by immunocytochemistry, showing similar expression patterns of CD44 in both control and CSC-treated MSCs in the absence or presence of HA (Fig. 3B).

### CSC inhibits both Cortactin and Survivin Expression levels parallel to CD44

**Figure 4:**
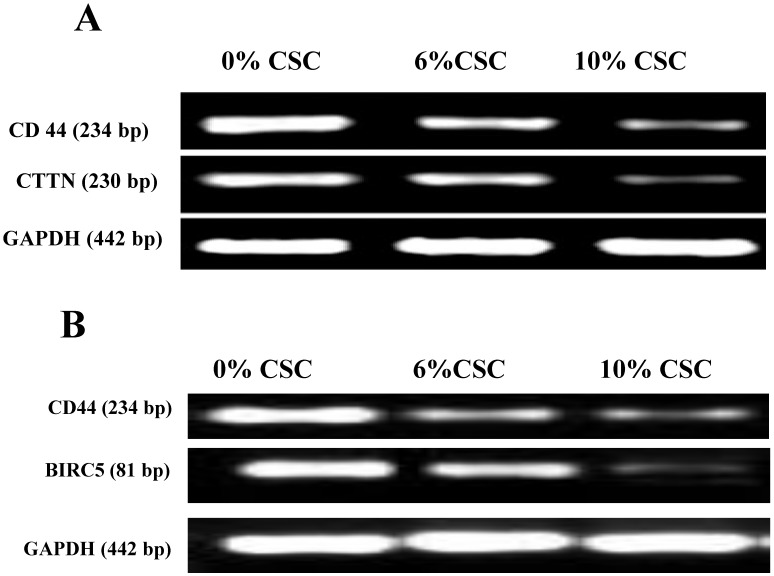
** CSC inhibits both Cortactin and Survivin RNA expression levels parallel to CD44 expression. (A)** CSC-reduced CD44 RNA levels paralleled CTTN RNA expression levels. **(B)** CSC-reduced CD44 RNA levels paralleled BIRC5 RNA levels. Shown are the cropped blot images representing indicated proteins, and all the gels from three separate experiments have been run under the same experimental conditions.

**Figure 5:**
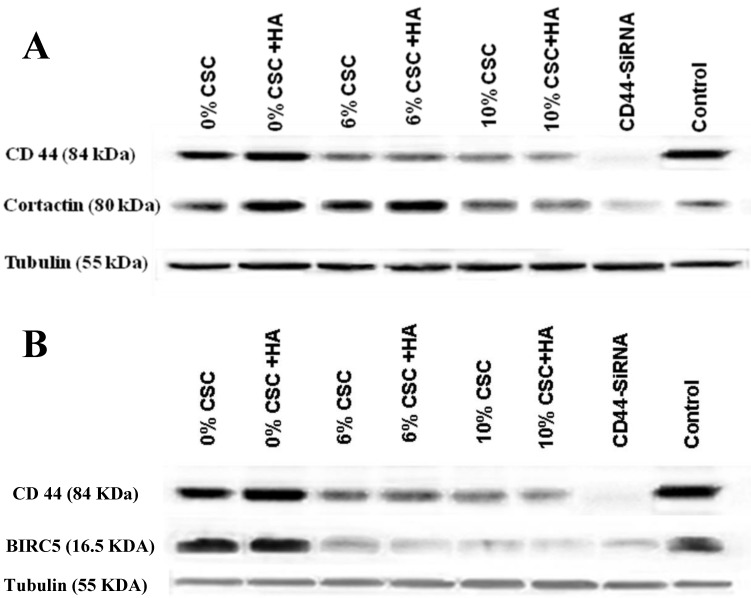
** CSC inhibits both Cortactin and Survivin protein expression levels parallel to CD44 protein expression. (A)** CSC-reduced CD44 protein expression levels paralleled CTTN protein expression levels. **(B)** CSC-reduced CD44 protein expression levels paralleled BIRC5 protein expression levels. Shown are the cropped blot images representing indicated proteins, and all the gels from three separate experiments have been run under the same experimental conditions.

We have recently discovered that CD44 can impair cancer cell migration and invasion *via* its two novel transcriptional downstream targets, CTTN 15, Survivin (BIRC5) 16. To test whether CSC-decreased CD44 expression also decreases its transcriptional targets, CTTN and BIRC5, we examined their respective RNA (Figure 4) and protein (Figure 5) expression levels, using RT-PCR and western blot analyses, respectively.

In fact, when CD44 expression levels were reduced both at RNA and protein levels in response to CSC treatment of MSCs, both CTTN and BIRC5 expression levels were reduced (Figures 4 and 5). More interestingly, inhibition of CD44 using specific CD44 siRNA primers showed the same patterns of inhibition of both CD44 transcriptional targets, CTTN and BIRC5 (Figures 4 and 5). Taking together, CSC-inhibited CD44 expression appears to directly affect its CTTN and BIRC5 downstream target signaling pathways in response to CSC treatment of MSCs.

### CSC impairs migration and invasion properties of MSC, mediated through CD44 signaling

**Figure 6:**
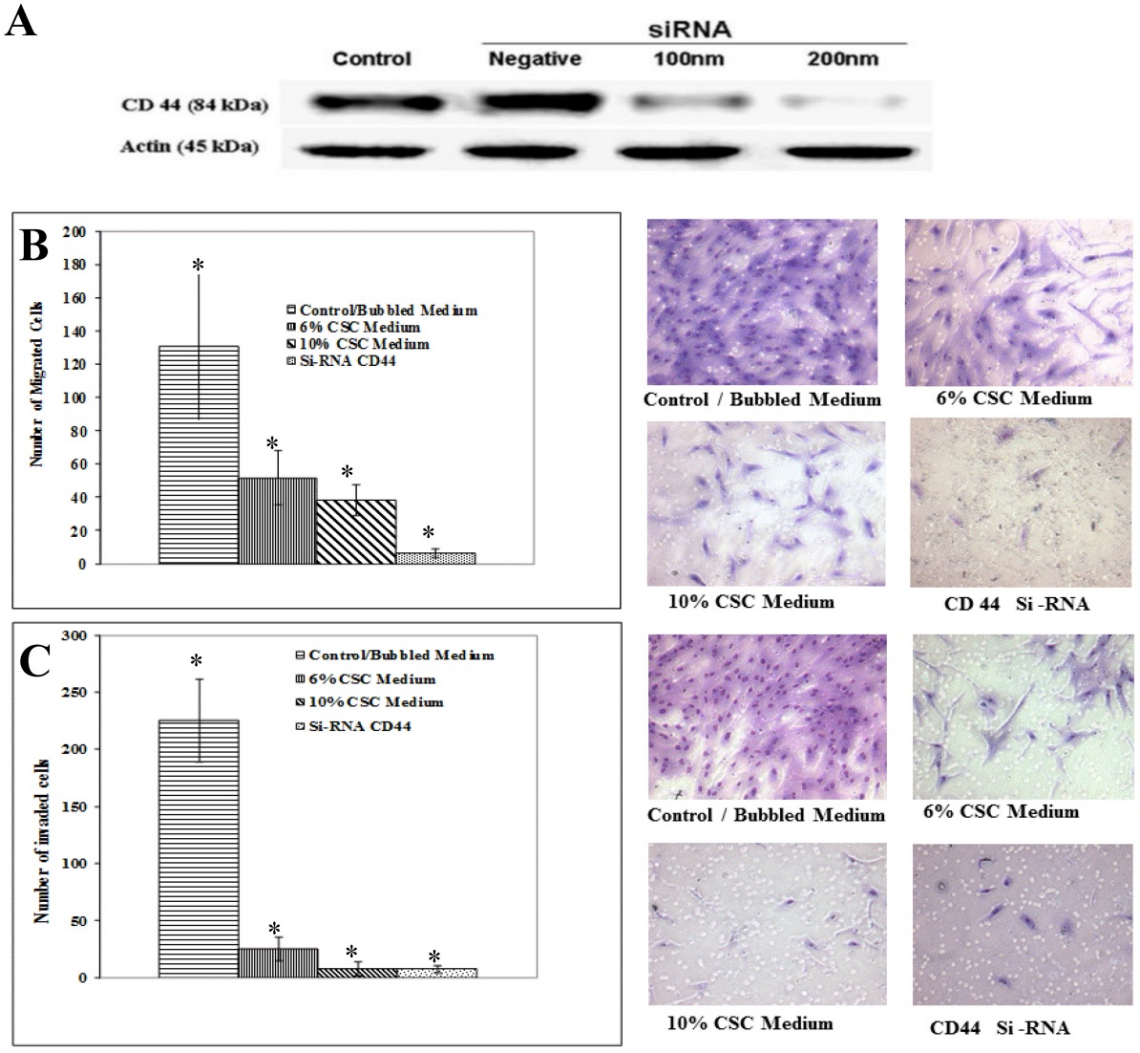
** CSC impairs migration and invasion ability of MSC mediated through CD44. (A)** Gradual suppression of CD44 expression using CD44 SiRNA. CD44 inhibition was optimized by different doses of siRNA. Shown are the cropped blot images representing indicated proteins, and all the gels from three separate experiments have been run under the same experimental conditions. **(B)** Representative images of Boyden chamber membranes represent the number of captured cells during migration, illustrating the difference in migrated cell numbers. **(C)** Graphical representation of invading cells was demonstrated by invasion assay. Note a marked decrease in MSC cell migration and invasion capabilities after inhibiting CD44 expression using siRNA. Control cells were cultured in the same conditions. Representative images of Boyden chamber membranes represent the number of captured cells during the invasion, illustrating the difference in invaded cell numbers. The difference is considered statistically significant (student's t-test *P<0.001).

To test whether CSC treatment of MSCs affecting CD44 signaling also affects its associated function in MSC migration and invasion, we initially optimized CD44 inhibition, using different doses of siRNA as shown in Figure 6A. The decrease of CD44 expression was dose-dependent of siRNA concentration.

Similarly to the experiments performed above, MSC Cells were treated with CSC (6%) and (10%) doses and with media containing siRNA specific to CD44. The Boyden chamber assay was carried out to measure both migration (absence of Matrigel) as well as MSC invasion (presence of Matrigel). In all cases, the results showed a marked decrease in migrated (Fig. 6B) and invaded (Fig. 6C). These results put together suggest that mobilization of MSC cells to damaged lung might be mediated by CD44 signaling pathways.

## Discussion

In this study, we examined the effect of cigarette smoke on CD44-mediated MSC migration and invasion. Our results showed a marked decrease in migrating cells after CSC exposure; this is consistent with a previous study demonstrating that CSC attenuated fibronectin and platelet-derived growth factor stimulated chemotaxis, accompanied by a detrimental effect on the regenerative capacity of MSCs 19.

Cigarette smoke may lead to cell death, inhibit MSC growth, and reduce their ability to migrate and engraft while increasing their senescence 19. It has also been reported that smoke interferes with the function and the normal processes in lung fibroblasts and bone marrow stromal cells 20,21. Our results showed a consistent temporal and quantitative relationship between CSC and MSC, especially at lower concentrations. Concentration-dependent decrease in proliferation of MSCs was observed over a period of 36 hours, suggesting that growth inhibition is unlikely induced by direct toxic effect. Interestingly, low doses of CSC stimulated proliferation, while higher concentrations decreased MSC proliferation.

CD44 is a transmembrane glycoprotein abundantly expressed on the surface of MSCs, and commonly used as a characteristic marker for MSC. CD44 is essential in the hematopoiesis process, tumor metastasis, lymphocyte activation, and MSC homing 10,11,22. It also serves as a link between cells and the extracellular matrix, regulating cell adhesion and migration 10,11,22. While HA is its major ligand, CD44 can also be activated by other ligands such as osteopontin, collagens and fibronectin 11,23-27.

Nicotine is one element of cigarette smoke that inhibits CD44 expression in a group of bone marrow stromal cells and in bone marrow derived-endothelial cell line (STR-12) 18,28,29. In our study, we found that in response to CSC, a concentration-dependent decrease in CD44 expression was observed at both mRNA and protein levels. Paradoxically, several studies have reported an increase in soluble CD44 in smokers, thus suggesting increased production and/or increased shedding 30-32. However, our data suggests a transcriptional level inhibitory effect, which is not consistent with shedding as the mechanism for reduced CD44 expression in our experimental setting.

There is a significant evidence supporting the role of CD44 in migration and homing of MSC; Zhu et al., 2006 have demonstrated that CD44 facilitated MSC adhesion and migration in murine CD44^-/-^ bone marrow stromal cells 33. Sackstein *et al*., demonstrated that induction of an alternative CD44 glyco-form on MSCs facilitated their homing 34, suggesting the involvement of CD44 in MSC motility. Our results support this finding, as RNAi inhibition of CD44 clearly suppressed migration and invasion of MSC.

HA is the major ligand for CD44 In emphysema, HA forms a complex with other GAGs to provide a protective shield that blocks access of CSC to molecules such as elastin, 35. HA also targets CD44 and inhibits FcepsilonR1 signaling that involves ROS and MAPK 18,36. Our results showed that CSC-induced reduction in CD44 expression was abolished when cells were treated with HA (Fig. 2). This confirms the protective effect that HA imparts against CSC-induced cell damage. Interestingly, smoking increases mucin production and mucus hypersecretion in a ROS-dependent manner 34. This aggravated mucus secretion in airway epithelia appears to be directly related to depolymerization of hyalouronan 34. Depolymerization of HA is pathologically an important process, since functions of HA are size-dependent, and low-molecular-weight HA is considered a “danger signal” 34.

We have previously demonstrated using Microarray analysis that CD44 promotes breast cancer cell invasions and metastasis to the liver 37, and identified CTTN 15 and BIRC5 37 as downstream target genes underpinning CD44-promoted cell survival, migration and invasion. While CTTN is a marker of motility, due to its close relation to the actin cytoskeleton, BIRC5 is an elusive protein required for the survival of the cells, and these two proteins play a key role in lung injury 16,38. The results of the present study revealed that CSC inhibited the expression of CD44, which in turn downregulated the expression of both its downstream transcriptional targets, CTTN and BIRC5. This parallel decrease of CTTN and BIRC5 was confirmed by RT-PCR, and further confirmed by RNAi inhibition of CD44 leading to significant reduction in CTTN and BIRC5 expression. Evidence supports a role of CD44 for homing of hematopoietic precursor cells and T-lymphocytes 39. A previous study by Sackstein *et. al.*, 2008, demonstrated induction of an alternative CD44 glycoform on MSC, which facilitated their homing to the bone, suggesting the importance of CD44 in MSC motility 34. Our results support this finding, as inhibition of CD44 with specific iRNA strongly suppressed migration and invasion of MSC cells (Fig. 4). Inhibition of CD44 with RNAi strongly suppressed migration and invasion of the MSC cells. This indicates the importance of CD44-downstream signaling in MSC motility 34. These data providing a clear evidence that CD44 signaling are vital for migration and invasion of MSC are supported by recent studies, emphasizing the regenerative potential of MSCs in the development of future cell-based clinical trials and therapies.

In summary, this study demonstrates the potential involvement of CD44 in the impairment of MSC migration and invasion in cigarette smokers (or in response to cigarette smoke). Furthermore, our findings suggest that this mechanism is mediated through a dual transcriptional regulation of CTTN and BIRC5 by CD44. These findings provide insights into potential novel mechanisms by which smoking may contribute to the development of osteoporosis, lung injury and emphysema, and further suggest that a caution must be taken before using smokers' MSC as source for cell therapy. In addition to a better understanding of the mechanisms by which CD44 mediates cigarette smoke-suppressed MSC cell motility, this investigation identified CD44/CTTN and CD44/BIRC5 signaling pathways as potential targets to pave the way towards the design of therapeutic strategies against cancer, emphysema and other CD44-associated diseases 40.

## Materials and Methods

### Cell Culture

Human bone marrow derived human MSC (MSC) were obtained from the Tulane Center of Gene Therapy (New Orleans, LA) and cultured in α-MEM medium containing 16.5% FBS (v/v, Atlanta Biologicals, Miami, FL), 2 mM L-Glutamine at 37°C with 5% carbon dioxide. The media was supplemented with dried sodium hyaluranon (Lifecore Biomedical, MN, U.S.A) at 100µg/ml concentration. All experiments were conducted with cells cultured to 60% confluence. Cigarette smoke condensate (CSC) was prepared by bubbling 40 ml of plain α-MEM with four 3R4F Reference Research Cigarettes (The University of Kentucky, College of Agriculture, Lexington, KY) at a negative pressure of 10 cm H_2_O, pH adjusted to 7.4 and filtered with 0.22 µm. This was used as 100% and diluted as desired in further experiments. The CSC was prepared fresh for each experiment and added to fresh media to achieve the desired concentration (final concentration of 5% FBS for all conditions). Control media was prepared in a similar fashion except room air was bubbled through the media.

### Antibodies, Chemicals & reagents

The following antibodies were used: Mouse polyclonal anti-CD44 (R&D systems), mouse monoclonal anti-CD44 (Santa Cruz Biotechnology, CA), rabbit polyclonal anti-GAPDH (Santa Cruz Biotechnology, CA), mouse monoclonal anti CTTN (Upstate cell signaling solution), rabbit monoclonal anti BIRC5 (Santa Cruz Biotechnology), goat anti-mouse IgG HRP CD44 (Santa Cruz Biotechnology), goat anti-rabbit IgG HRP CD44 (Santa Cruz Biotechnology) antibodies.

### Proliferation Assay

The AlamarBlue® cell proliferation assay (Invitrogen, Carlsbad, CA) was utilized to determine the toxicity of CSC on MSC. MSC were seeded at 2x10^4^ cells on Costar black tissue culture plates and allowed to attach overnight. The cells were then incubated in media containing various doses of CSC and AlamarBlue® was added to all the wells. Cells were observed at serial time points for morphological changes and fluorometric readings were taken at excitation of 540 nm and emission 610 nm.

### Cell Migration and Invasion Assays

Migration and invasion chambers were prepared by coating cell culture inserts (12 µm pore size, Millipore, MA) with 200 µg/ml of Matrigel™ (BD Biosciences, MA), which serves as a basement membrane substitute corresponding to the basement membrane of blood vessels. Assays were conducted using MSC exposed to CSE, control media as well as with media containing RNAi of CD44 for four hours as previously described 40. After trypsinization cells were resuspended in α-MEM with 1% FBS at 5 x10^5^ cells/200µl/well, added to the upper chamber and then incubated for 24-hrs at 37°C. After 4 hours, the cells were detached and seeded into transwell membranes (pore size-12µm) with serum deficient media placed in 12 well plates containing 1% FBS containing MEM -alpha medium. The 12 well plates were incubated for 24 hours at 5% CO_2_ and 37°C. At the end of 24 hours, the upper surface of the filter was then wiped with a cotton swab to remove non-migratory cells. The cells attached to the other side of filter were stained with a Diff-Quick stain set (Dade Behring Inc.), photographed under a phase-contrast microscope and the total number of stained cells for each filter was tabulated using a counting grid.

### Western blotting

Cell lysates were prepared from cells treated for 24 hours with bubbled CSC or control media and the final protein concentration in the supernatant was determined using the Bradford Protein Assay Reagent (Sigma-Aldrich, St. Louis, MO) as previously described 40. Samples (60µg) were boiled for 5 minutes in an equal volume of reducing buffer and resolved on 12% polyacrylamide gels and electroblotted onto PVDF membranes. Membranes were probed with mouse anti-CD44 (1:2000 dilution, R& D systems), mouse monoclonal anti CTTN antibody (1:2000 dilution, Upstate cell signaling solution), rabbit monoclonal anti BIRC5 antibody (1:2000 dilution, Santa Cruz Biotechnology)and goat anti-GAPDH antibody (1:2000 dilution, Santa Cruz, CA) and detected with a goat anti-mouse and donkey anti-goat IgG-HRP (1:5000 dilution, Santa Cruz, CA) using chemiluminescence (Supersignal West Femto, Pierce).

### Preparation of RNA samples

Cells were treated with CSC and harvested after 12 and 24 hours. RNA was isolated using RNeasy Mini kit (Qiagen) according to the manufacturer's recommendations. Isolated RNA samples were assessed for quantity and purity (NanoDrop Technologies Inc. DE, USA) and stored at -80 ^0^C for further analysis.

### RT-PCR Analysis

For RT-PCR analysis (Qiagen One Step RT-PCR kit), 1.0 µg of total RNA was reverse transcribed using standard reagents according to the manufacturer's instructions. Samples were incubated in the PTC-200 Thermal Cycler for reverse transcription at 50°C for 30 min, initial PCR activation step at 95°C for 5 min followed by 32 polymerase chain reaction cycles. Each cycle consisted of at 95˚C for 30 seconds, 56˚C for 30 seconds, and 68˚C for 1 minute. The final completion step was carried out at 68˚C for 10 minutes. The annealing temperatures for various genes were calculated according to their sequence and were optimized. Accordingly, the temperatures we used were: 55˚C for CD44 and CTTN, 51˚C for BIRC5 and 58˚C for GAPDH.

The following oligonucleotide primers were used: CD44: TTTGCATTGCAGTCAACAGTC (sense) and TTACACCCCAATCTTCATGTCCAC (antisense); CTTN: AAAGCTTCAGCAGGCCAC (sense) and TTTGGTCCTGTTTCAAGTTCC (antisense); BIRC5: AGCCCTTTCTCAAGGACCA (sense) and TCAATCCATGGCAGCCAG (antisense); GAPDH: ACCACAGTCCATGCCATCAC (sense) and TCCACCACCCTGTTGCTGTA (antisense). The RT-PCR products were examined by electrophoresis in 2% agarose gel containing 0.2 μg/ml ethidium bromide.

### RNAi-Mediated Depletion of CD44

Specific human CD44 siRNA oligonucleotides (5'-GGAAAUGGUGCAUUUGGUGtt-3' and 3'-CACCAAAUGCACCAUUUCCtt-5') and a Silencer® Negative Control #1 siRNA were synthesized commercially (Ambion, TX). Cells were seeded and grown to 50% confluency, washed twice in sterile PBS, then incubated with a transfection cocktail comprised of OPTI-MEM, Lipofectamine-2000 (Invitrogen) and the siRNA or scrambled oligonucleotides, at final concentrations of 100 and 200 nM for 24 hours at 37°C. Next day, cells were treated once more with the siRNA cocktail. Transfected cells were incubated at 37°C for an additional 24 hours before harvest and were then used in experiments. Depletion of CD44 expression in cells was confirmed by immunoblotting.

### Statistical Analysis

Differences between mean and standard deviation were assessed for statistical significance using one-way ANOVA with Newman-Keuls Multiple Comparison Test (GraphPad InStat version 5.00, GraphPad Software, CA). *p* value less than 0.05 was considered significant.
